# Nanoclay gels promote re-epithelialization and improve the quality of skin wound healing in a diabetic mouse model

**DOI:** 10.1038/s41536-026-00495-6

**Published:** 2026-07-23

**Authors:** Daniel J. Page, Zilian Fan, Claire E. Clarkin, Rajopal Mani, Yang-Hee Kim, Jonathan I. Dawson, Nicholas D. Evans

**Affiliations:** 1 Centre for Human Development, Stem Cells and Regeneration, Human Development and Health, Institute for Life Sciences, University of Southampton Faculty of Medicine, Tremona Road, Southampton, UK; 2 Bioengineering Sciences Group, University of Southampton, Southampton, UK; 3 School of Biological Sciences, Faculty of Environmental and Life Sciences University of Southampton, Southampton, UK; 4 Department of Medical Physics, University Hospital Southampton NHS Foundation Trust, Southampton, UK

**Keywords:** Biotechnology, Materials science

## Abstract

There is evidence that sorptive clays have been used since prehistory as materials to treat skin abrasions and wounds. Despite this, there is a paucity of studies that systematically address the efficacy of defined clays in improving skin wound healing. In this study, we tested the hypothesis that a well-defined synthetic smectite clay, Laponite, enhances healing in a delayed skin wound healing model. Full-thickness skin wounds were made in the back skin of *db/db* and wild-type male adult mice (8–10 weeks), and Laponite clay gels or controls of phosphate-buffered saline (PBS) or alginate were applied after 24 h and held in place with a semi-occlusive dressing. Although Laponite treatment did not accelerate macroscopic wound closure, it significantly improved healing quality, including re-epithelialisation, epithelial cell division, epithelial thickness and fibroblast invasion, compared with PBS- or alginate-treated *db/db* mice. Moreover, hair follicle anagen was stimulated in proximity to Laponite-treated wounds, but was absent in PBS- or alginate-treated wounds. Neutrophil infiltration at day 18 was also reduced. In contrast to alginate, in which VEGF improved wound healing, VEGF in Laponite showed negligible additional benefit. These data indicate a role for synthetic nanoclay gels in wound healing.

## Introduction

Clays have played an important role since prehistoric times in medicine. Clay-rich materials have been eaten to reduce the effect of ingested toxins, are used as haemostatic agents, and are widely used as active ingredients in medicines such as antacids, anti-diarrhoeic agents, topical skin salves or as anti-inflammatories^1,2^.

At the molecular level, clays have two principal units—tetrahedral and octahedral repeating subunits that assemble into sheet-like layers by virtue of their charge distribution^3^. The arrangement of these sheets depends on their composition and affects their physical properties. One example of a clay widely explored for biomedical applications is LAPONITE® XLG (herein Laponite), a synthetic clay based compositionally on the naturally occurring hectorite with the formula (Na^+^_0.7_[(Mg_5.5_Li_0.3_Si_8_O_20_(OH)_4)_]^−^_0.7_). The sheets of this clay delaminate to form nano-sized platelets, approximately 25 nm in diameter and 1 nm in thickness with a permanent negative charge on the platelet surface and a positive rim charge at <pH 9. These platelets form colloids in aqueous solutions with macroscale properties of viscoelasticity and thixotropy.

The high surface area (~370 m^2^/g) and charge distribution permit a very high capacity for the adsorption of biomolecules (>1 mg protein/ mg nanoclay)^2,4^ making them an attractive biomaterial for drug delivery applications. They are currently under development as a potential method of delivering osteogenic agents for bone repair^5^ and have shown potential for localised delivery of vascular endothelial growth factor (VEGF) to promote angiogenesis^6^.

The interest of clays for wound healing is attested by the large number of recent reviews on the subject^7–10^. Despite this interest, there is a paucity of experimental studies directly examining clays in wound repair using controlled in vivo models. Studies to date have assessed various types of clay in excisional wounds in healthy rats, and report improvements in the rate and thickness of re-epithelialisation as well as changes in other measures such as angiogenesis and collagen deposition^11–16^.

Given the well-documented sorptive properties of clays, chronic wounds represent a particularly promising context in which to investigate clay-based treatments. Chronic wounds, including lower extremity ulcers that are a significant risk for diabetic patients, are characterized by excessive levels of inflammatory cytokines, proteases, and reactive oxygen species, all of which contribute to immune cell accumulation (such as neutrophils and pro-inflammatory macrophages). This prevents the progression of wound healing from the inflammatory phase to the regenerative phase^17^, resulting in delayed healing and impaired tissue regeneration^18,19^. The highly sorptive capacity of clays in this context, together with their potential for delivering pro-healing agents such as growth factors, suggests potential for modifying the chronic wound environment. Laponite is also attractive in this context for its ability to generate stable, hydrated colloidal gels supportive of cell migration and angiogenesis – processes that are again significantly retarded in chronic wounds^20,21^.

In this current study, we tested the hypothesis that topical application of Laponite clay gel could promote wound healing and skin regeneration in a male *db/db* mouse model of delayed wound repair.

## Results

### Laponite treatment did not accelerate wound closure in *db/db* mice

To confirm physiological differences between *db/db* and wild type mice during wound healing^22^, we made 8 mm wounds in the back skin of mice and measured body mass, blood glucose concentration and wound closure with respect to time (Fig. S1). These results prove that *db/db* mice had a significantly greater mass (~40%), higher blood glucose levels than healthy mice (*p* = <0.0001). and exhibited delayed wound closured compared to healthy mice.

**Fig. 1:**
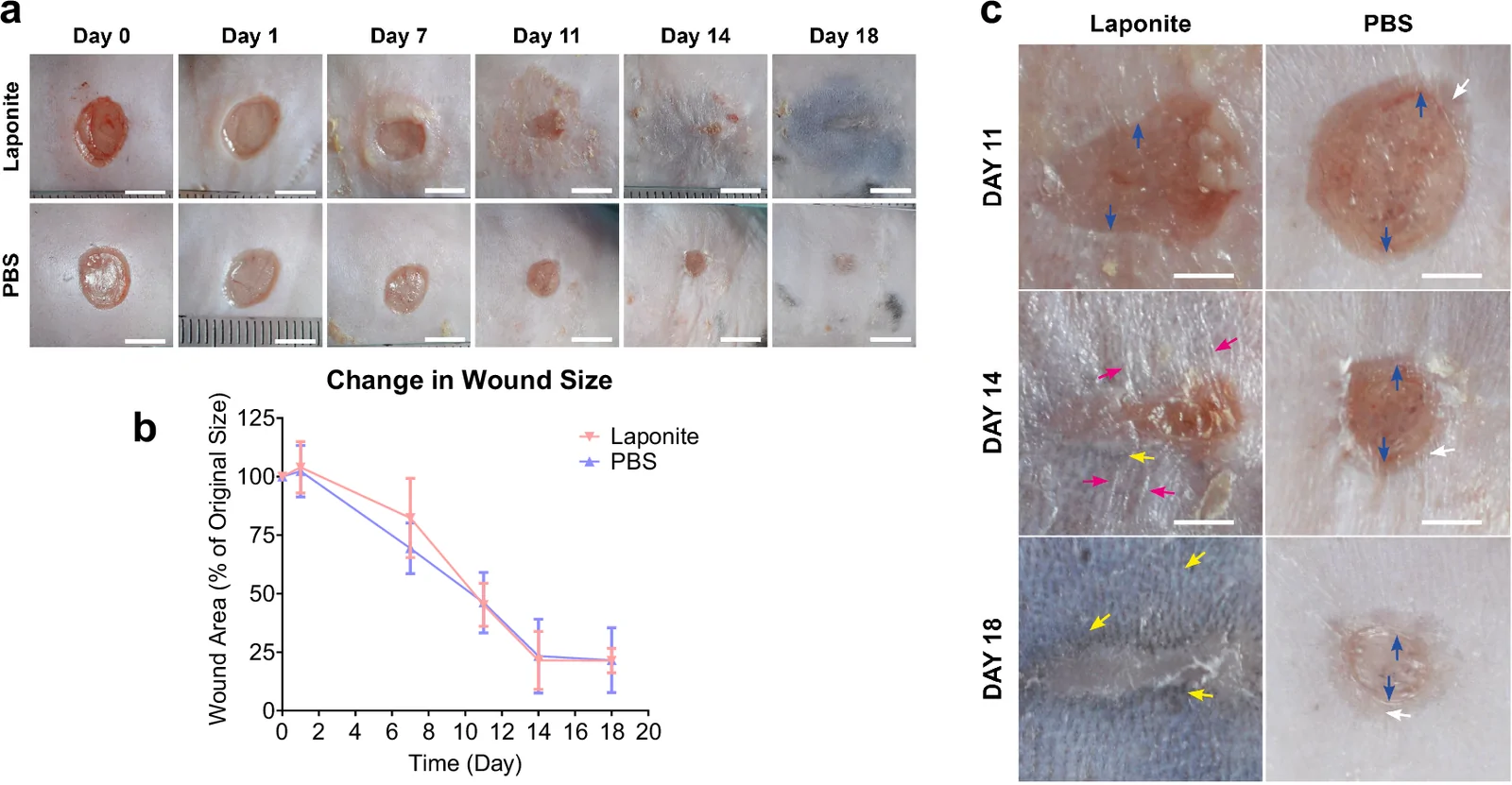
Laponite does not enhance the rate of wound closure in *db/db* mice. Macroscopic imaging of wounds (**a**) and quantification by image analysis (**b**) indicate negligible differences in wound closure in clay gel vs. PBS treated wounds in *db/db* mice (*n* = 9 mice days 0 and 1; *n* = 8 mice day 7, *n* = 6 mice day 11 and 14; *n* = 3 day 18; each n corresponds to the is the mean of 2 technical repeats; see “Methods”). **c** Inspection of wounds indicated a more mature, fibrous appearance in clay gel treated wounds compared to control between day 11 and 18, in addition to a dark speckled pigmentation indicative of hair follicle anagen only in former groups (hair follicle anagen—yellow arrows; wrinkling—pink arrows; epithelial migration—white arrows; wound edge—blue arrows). Error bars = SD.

Next, to test the hypothesis that a Laponite clay gel improves wound healing in this model, we applied Laponite gels to skin wounds and wound photographs were taken over an 18-day period to compare differences in wound size and colour at different sites treated with Laponite or PBS (Fig. 1a). We found PBS-treated wounds had a slight, though not significant acceleration of wound closure for the first 7 days and after 7 days, there were no apparent differences between the two treatments.

Macroscopic wound appearance was further assessed to determine the progress of wound healing (Fig. 1). Residual wound exudate left a pink-coloured impression around the wound periphery (Fig. 1a; day 7 and 11 Laponite images). By day 14, in central wound regions, Laponite-treated wounds showed a marked reduction in redness compared to PBS treatments (Fig. 1c). Healthy tissue adjacent to the Laponite wound was now grey with a speckled pigmented appearance as opposed to red in the case of PBS treatment, likely due to hair follicle anagen. PBS-treated wounds on day 14 had a depressed central region which appeared redder in colour than adjacent tissue and which had shrunk significantly compared to Day 0. This central region most likely comprised wound granulation with the partial exposure of subcutaneous tissue. Beyond the circular depression region of PBS wounds and towards the margin of the original defect, there was evidence of soft tissue formation, indicative of epithelial migration. In Laponite-treated wounds, wound contraction was more pronounced and less red subcutaneous tissue was exposed, along with the growth of new tissue at the edge of the wound bed, indicating partial wound healing.

By day 18, Laponite wounds exhibited negligible redness, darker grey margins and reduced dehydration, suggesting increased collagen deposition and re-epithelialisation (Fig. 1c). PBS wounds also improved but retained a redder, open appearance compared to Laponite. A depressed inner circle region persisted which appeared covered with a thin tissue layer. Although the closure rates were comparable (Fig. 1b), in PBS this appears to be due to increased contraction whereas Laponite-treated wounds exhibited a more mature, fibrous appearance.

### Re-epithelialisation rate and epithelial thickness in *db/db* mice is increased by Laponite treatment

**Fig. 2:**
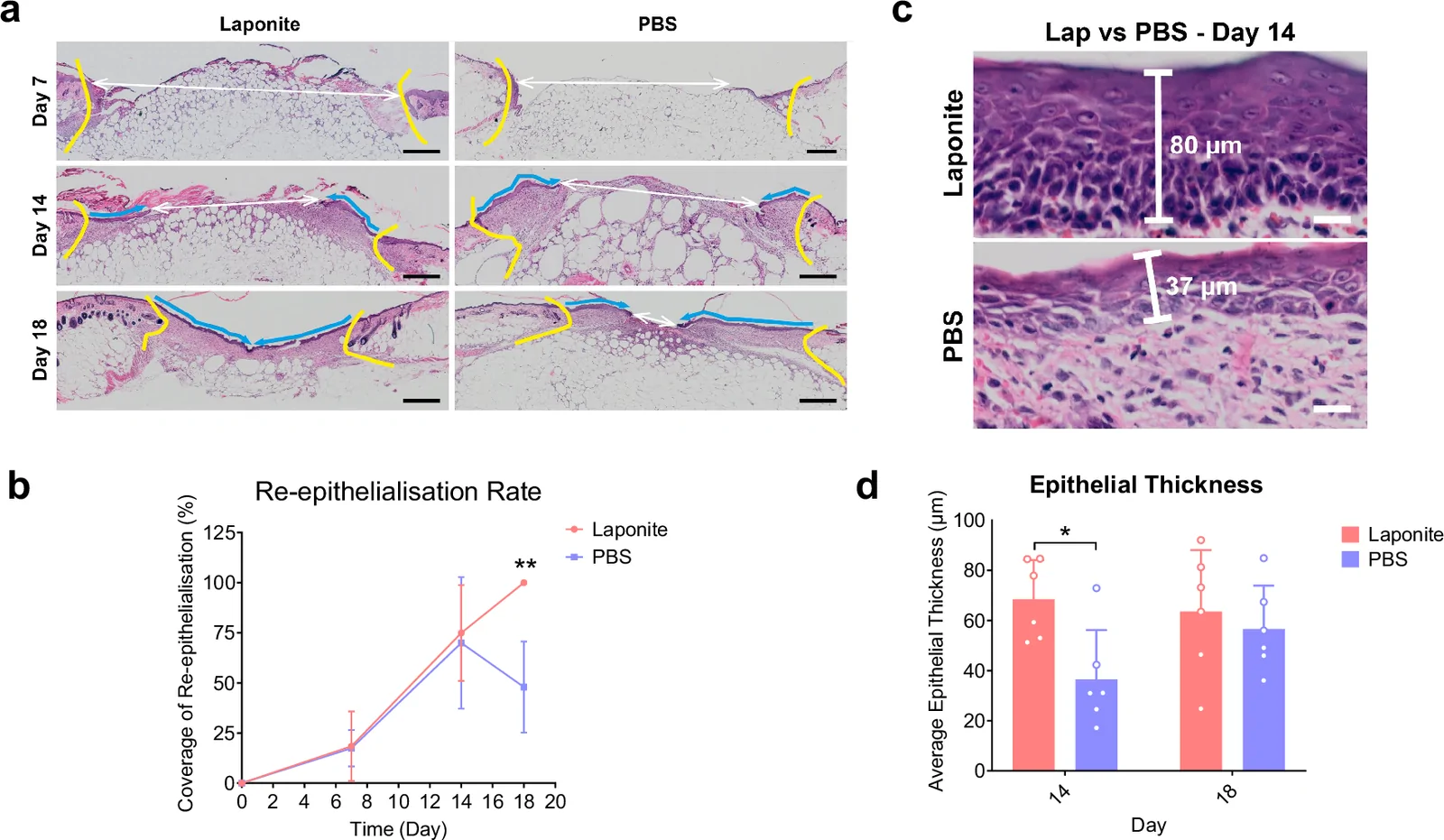
Laponite improves re-epithelisation in *db/db* wounds. **a** H&E-stained tissue sections of wounds showing re-epithelialisation between day 7 and day 18; yellow lines = wound margins from original defect; white arrows = open wound and blue arrows = reepithelialisation coverage. **b** At day 18 all clay gel-treated wounds had fully re-epithelialised whereas PBS-treated wounds had not (**p = <0.01). *n* = 3 (*db/db* mice). **c** Clay gel-treated wounds exhibited greater thickness of newly-formed epithelium with (d) a significant increase at day 14. (*p = <0.05). *n* = 3 (*db/db* mice). Scale bars = 500 µm.

Re-epithelialization primarily occurs during the proliferative phase of wound healing^23^. To evaluate re-epithelialisation during wound healing, we measured the rate of re-epithelialization and the thickness of the newly formed epithelium. Wound tissue was harvested at day 7, 11 and 18 post-surgery, serially sectioned and stained with haematoxylin and eosin (H&E) (Fig. 2a). By day 18 all Laponite-treated wounds had fully re-epithelialised whereas PBS-treated wounds had not (Fig. 2b , *p* < 0.01).

Epidermal thickness was measured at multiple points in Laponite-treated compared to PBS treated wounds (Fig. 2c). At day 14 epidermal thickness was significantly greater in Laponite treated wounds (~10 cell layers) compared to PBS treated wounds (~ 5 cell layers) (Fig. 2d, *P* < 0.05). Basal layer cells in Laponite appeared larger with darkly stained nuclei, while at more distal cell layers cells appeared more stratified. At 18 days, however, there was no statistically significant difference in this metric.

### Laponite treatment reduces neutrophil persistence and enhances cell proliferation within the wound

**Fig. 3:**
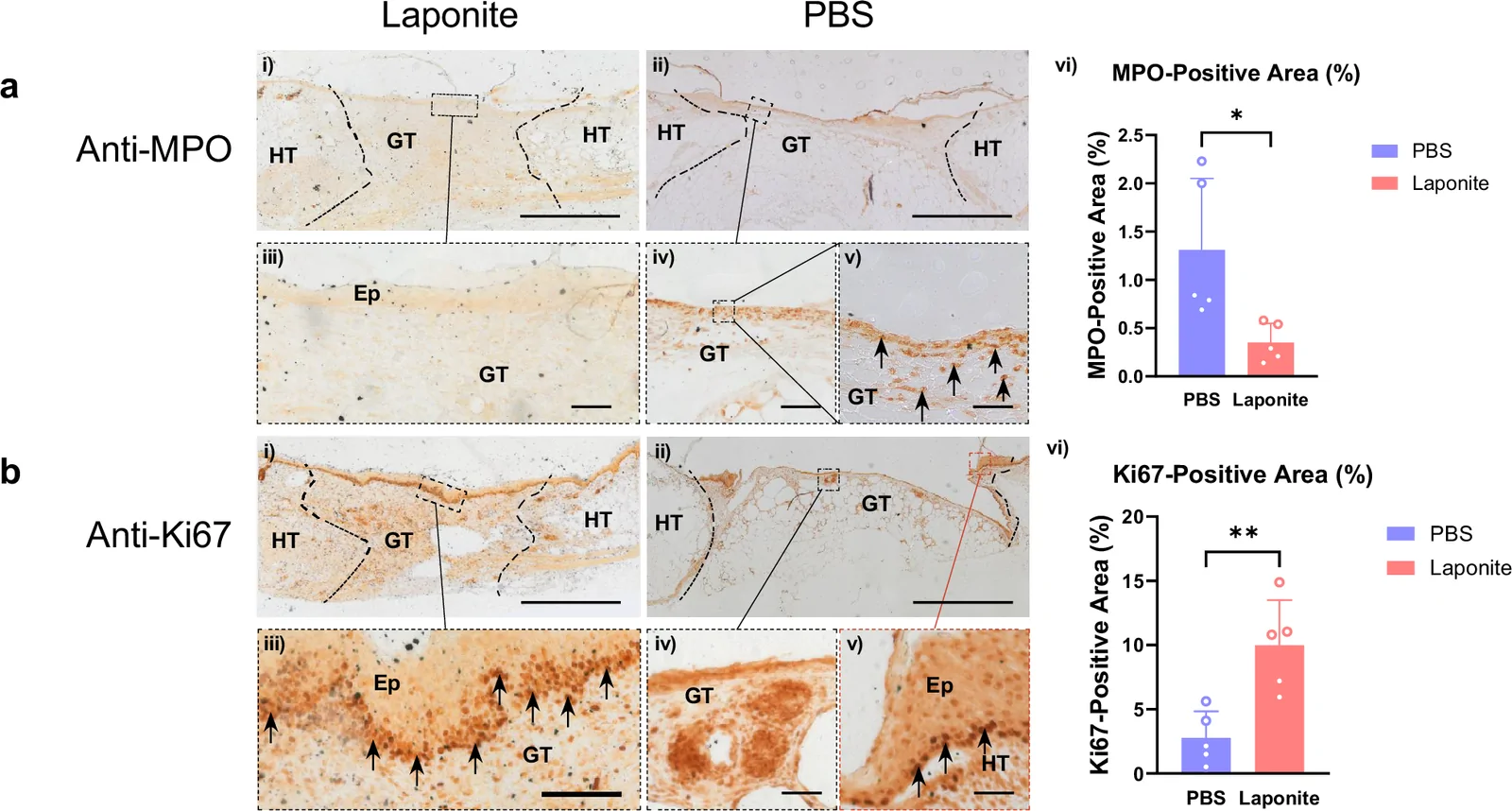
Laponite wound treatment reduces neutrophil number and increases epithelial cell proliferation. **a** There was no evidence of inflammatory neutrophils present within clay gel-treated tissue after 18-days post-surgery (i and iii). In contrast, PBS-treated wounds exhibited neutrophil activity within the wound bed indicative of a wound still within an inflammatory stage of wound repair (ii, iv and v). (vi) Quantifications (*n* = 5). *p < 0.05. Error bars = SD. **b** Newly-formed epithelium within clay gel-treated wound shows evidence of Ki67 positive cells in the basal layer of the epidermis (i and iii) which was less evident in PBS-treated wounds (ii) although Ki67 positive cells were present in the epithelial tongue at the wound margins (v). (vi) Quantifications (*n* = 5). **p < 0.01. Error bars = SD. i and ii scale = 1 mm, iii scale = 200 µm, iv and v scale = 100 µm. Dotted line = wound margin; Black arrows = positively stained neutrophil (**a**) or cell undergoing proliferation (**b**), Ep epithelium, GT granulation tissue, HT Healthy tissue.

The persistent presence of neutrophils indicates a prolonged inflammatory phase, which may impede the progression of wound healing^24^. Notably, no neutrophils were detected in Laponite-treated wounds at 18 days post-surgery (Fig. 3a, i and iii), suggesting resolution of inflammation. In contrast, PBS-treated wounds exhibited persistent neutrophil activity within the wound bed, indicating that these wounds remained in the inflammatory phase of the healing process (Fig. 3a, ii and iv).

Laponite-treated wounds also showed evidence of highly proliferative cells towards the basal layer, indicative of mature epithelial tissue (Fig. 3b, i and iii). In contrast, PBS wounds lacked newly formed epithelium (Fig. 3b, ii and iv) and showed highly-proliferative cells only at the wound margins (Fig. 3b, v). These observations suggest that Laponite enhances re-epithelialisation and promotes cell proliferation.

### Laponite gels stimulates hair follicle anagen in *db/db* mice

**Fig. 4:**
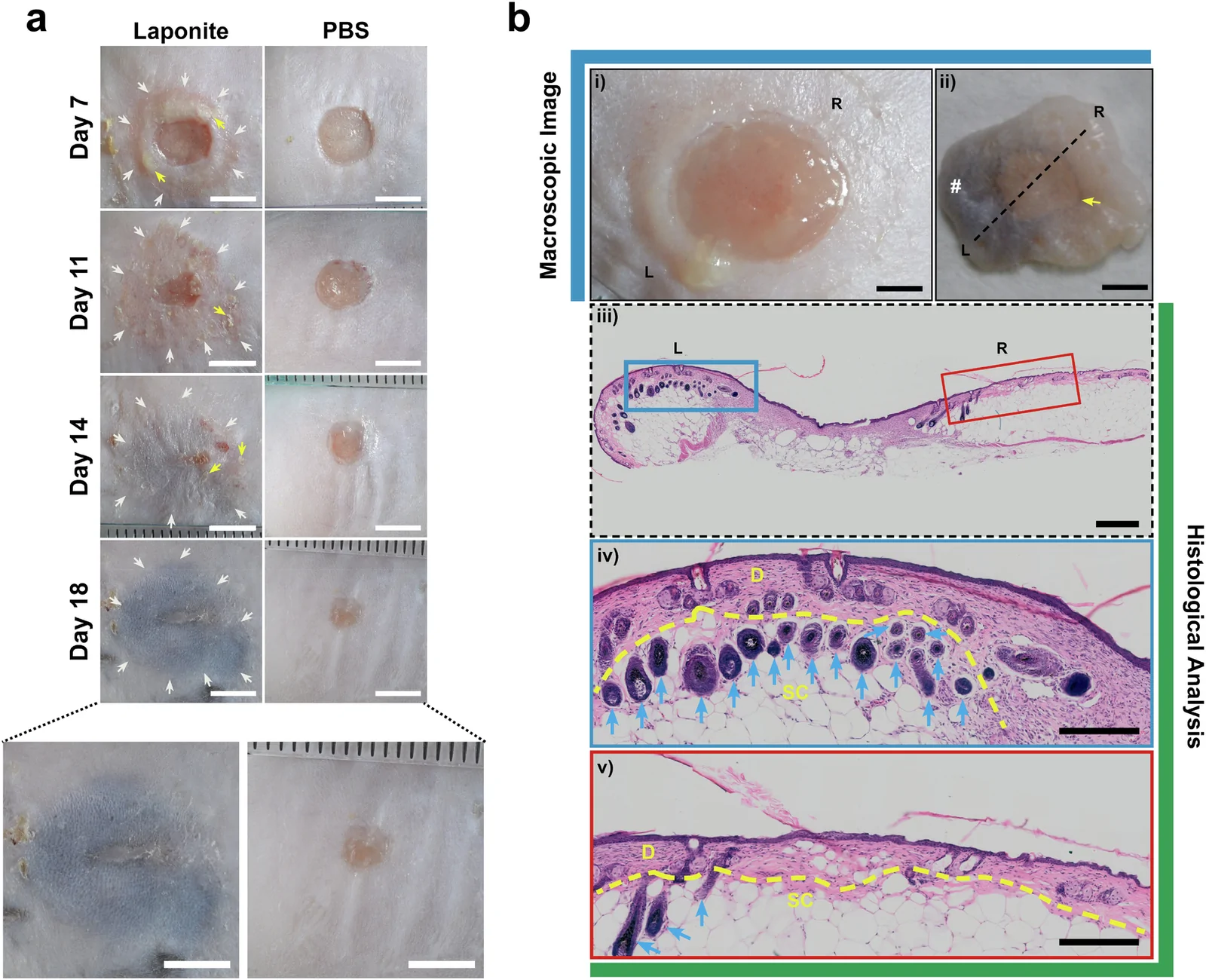
Wound exudate localised by Laponite gels stimulates hair follicle anagen. **a** Clay gel and exudate (yellow arrows) localises around the wound periphery at 7 days (demarcated by white arrows) and from day 14 is replaced by a dark ‘ring’ suggestive of hair follicle anagen (left panels), whereas this was not evident in any of the PBS-treated wounds (right panels). Scale bars = 5 mm. **b** Another wound shown at d7 in (i) was harvested at day 18 (ii) and processed, sectioned and stained for H&E. (Scale = 2 mm). (iii) A tissue cross-section taken approximately at the location depicted by the dotted line in (ii), with the orientation of the tissue shown by left (L) and right (R) (Scale = 500 µm) (iv) Higher magnification image of the right region (red box in iii), and (v) the left region (blue box in iii) (Scale = 200 µm); there was a greater number of hair follicles present in the left side than the right side. A high proportion of the hair follicles existed within the subcutis (blue arrows) which is indicative of hair follicles undergoing anagen. Yellow dotted line = dermis/subcutis boundary; D dermis, SC subcutis.

Hair follicle pigmentation was evident within Laponite-treated wounds, extending several millimetres from the wound periphery. This pigmentation closely corresponded with areas of residual wound exudate (Fig. 4a, white arrows) suggesting a potential link between the presence of wound exudate and hair follicle growth via an anagen-inducing effect.

To investigate this, cross sections of this region were analysed with H&E staining (Fig. 4b i and ii). The regions where exudate was present had a higher density and area of hair follicles (Supplementary Fig. 2), many within the subcutaneous layer (Fig. 4b iv, blue arrows) indicative of anagen phase growth. In contrast, in the region without apparent exudate exposure (Fig. 4b i) only a couple of deep residing hair follicles were observed (Fig. 4b v, blue arrows). These data suggest that Laponite stimulates hair follicle anagen, likely through an exudate-mediated mechanism.

### Observations of Laponite integration/exclusion within Laponite-treated wounds in *db/db* mice

Based on the above results, we sought to clarify whether Laponite was integrated into or excluded by the wound bed. Auramine O, a selective marker for Laponite (Supplementary Fig. 3), was used to detect and track Laponite within the wound bed over the course of healing.

**Fig. 5:**
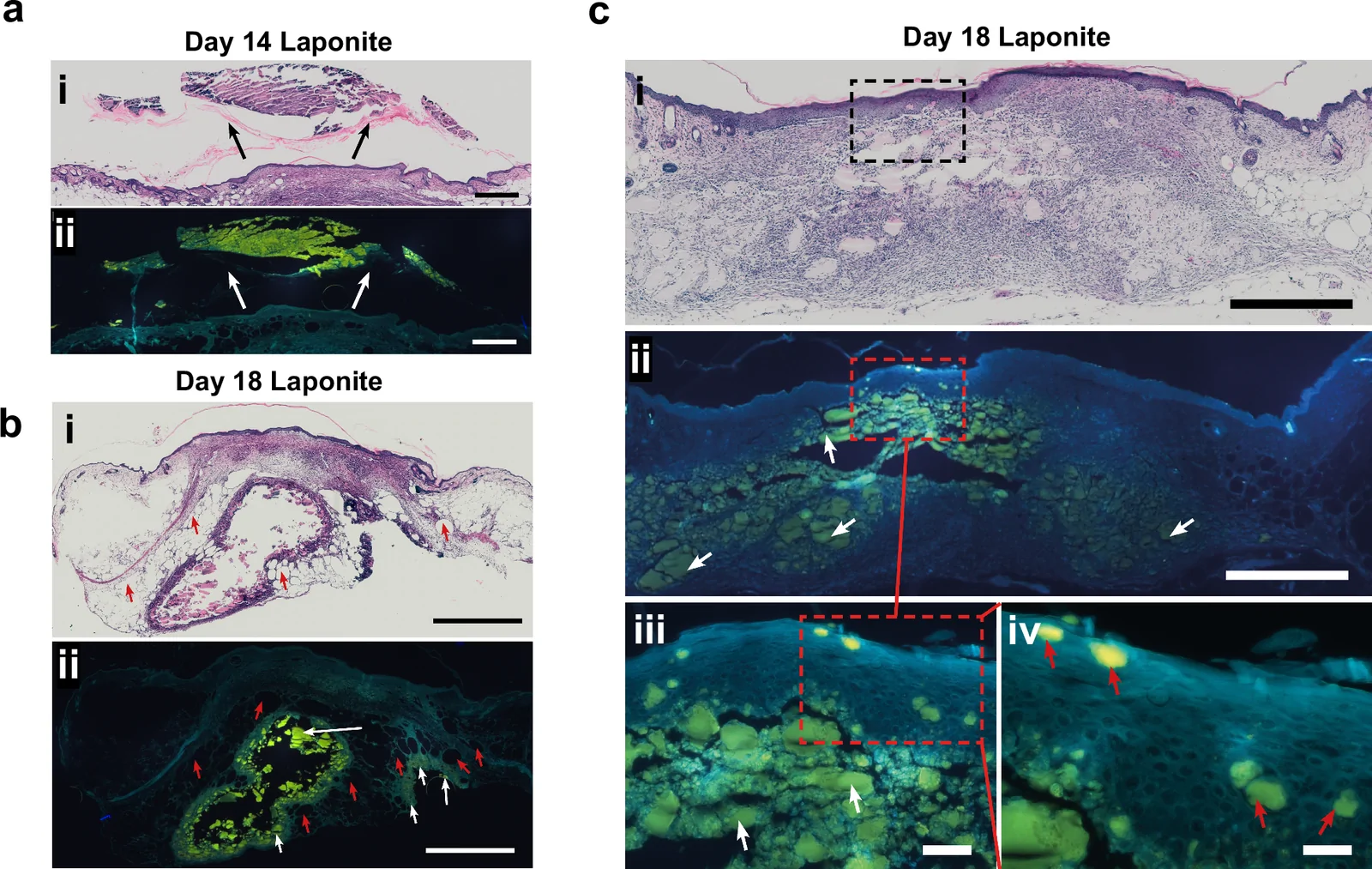
Laponite is incorporated into the tissue of healing wounds. **a** A fully re-epithelialised clay gel-treated sloughed clay gel from the wound surface (shown stained for H&E (i) and Auramine O (ii)). Scale = 500 µm. **b** In another wound, clay gel was incorporated into the subcutaneous tissue. Scale = 1 mm. White arrows = clay gel; red arrows = adipose tissue. **c** In some wounds clay gel was incorporated into the granulation tissue of the fully-re-epithelialised wound in addition to in the subcutaneous tissue. (i) shows H&E and (ii)-(iv) Auramine O staining of selected areas; scale = 1 mm for (i and ii); 500 µm for (iii) and 250 µm for (iv) White arrows = clay gel positively stained with Auramine O, red arrows = clay gel embedded within epithelial tissue.

By day 14, excluded Laponite (stained with the fluorescent marker Auramine O) was observed as a mass containing wound debris, separated from fully re-epithelialised wounds (Fig. 5a). However, some fragmenting Laponite gel persisted within the wound environment for up to 18 days post-surgery associated with granulomatous tissue responses. Figure 5a ii and b shows residual Laponite (white arrows) within the adipose tissue of the subcutaneous layer of the skin (red arrows) even after both wounds had fully re-epithelised. Interestingly, persistence of Laponite sometimes also occurred within the newly formed epithelium (red arrows) (Fig. 5c).

### Exogenous VEGF enhances alginate-mediated repair but does not further improve the pro-healing effect of Laponite

VEGF delivered via Laponite subcutaneously has been shown to significantly increase blood vessel formation^6^. As blood vessel formation (angiogenesis/vasculogenesis) is important in wound healing^25^, we hypothesised that delivery of VEGF using Laponite at the wound surface may offer further improvements to wound healing quality and closure rates.

**Fig. 6:**
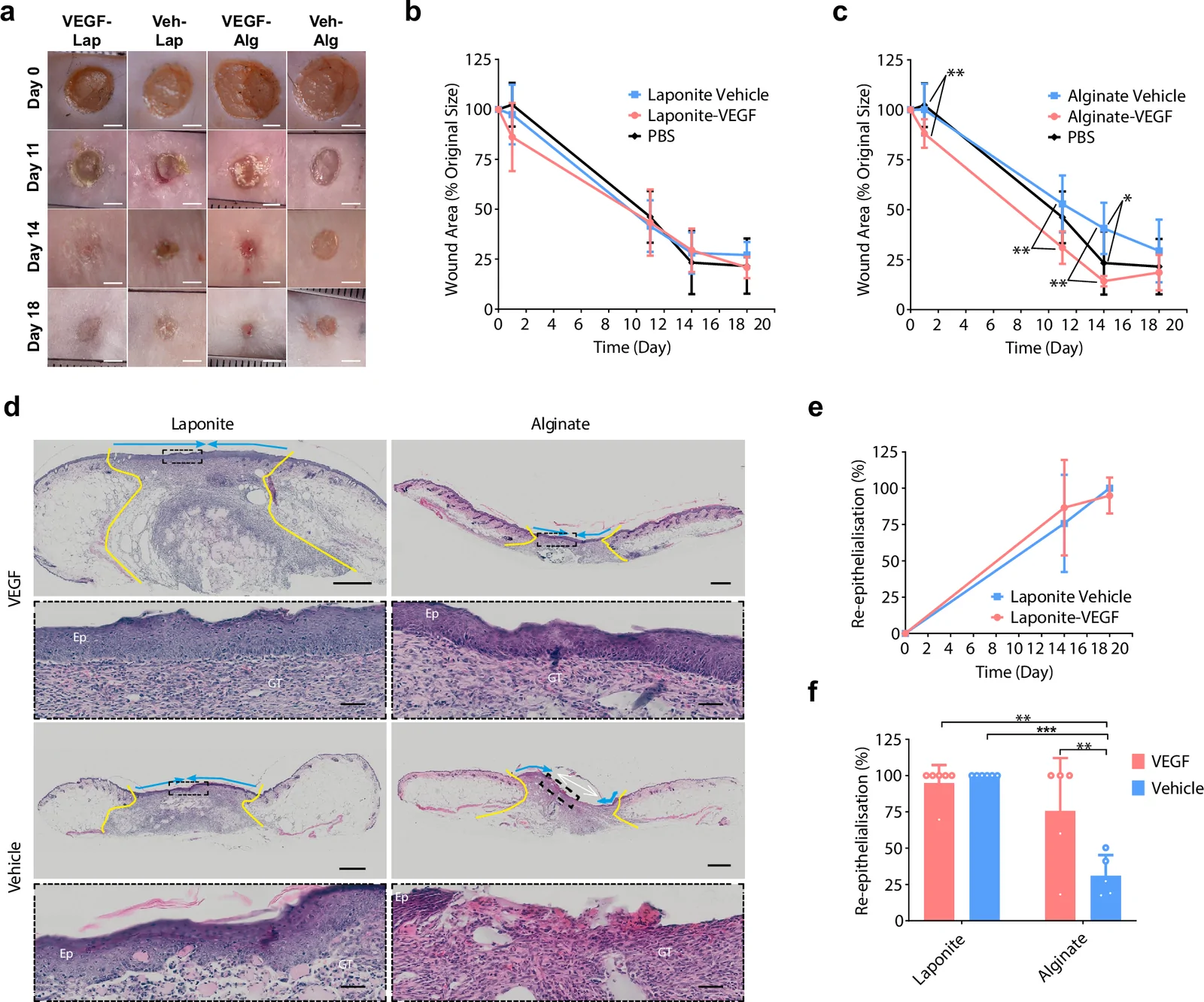
VEGF addition does not further enhance wound healing by clay gels. **a** Near to full closure was observed in all wounds treated with Laponite irrespective of VEGF addition whereas in the absence of VEGF the alginate vehicle only wound still exhibited granulated appearance. Scale bars = 2 mm. Differences in wound closure rates between Laponite (**b**) and alginate (**c**) treated wounds (±VEGF 40 µg/ml). *n* = 18 (*db/db* mice) **d** H&E-stained tissue sections of day 18 wounds that had been treated with 40 µg/ml VEGF encapsulated within Laponite gels and alginate gels; yellow lines = wound margins from original defect; white arrows = open wound and blue arrows = re-epithelialisation coverage; Ep = epithelium, GT = granulation tissue. **e** The rate of re-epithelialisation between Laponite-VEGF and vehicle Laponite showed minor differences over 18 days. *n* = 3 (*db/db* mice). **f** Percentage re-epithelialisation after 18 days. *, ** and *** denotes that *p* = <0.05, <0.01 and <0.001 respectively. *n* = 6 *db/db* mice. Error bars = SD.

Figure 6a shows photos of wounds taken from day 1 to day 18 following treatment with Laponite or alginate gels (± VEGF) 1-day post-surgery. From day 0 to day 11, all wounds had differences in appearance. By day 11, alginate treated wounds had a white precipitate within the wound bed and exhibited a redder appearance than Laponite-treated wounds, which appeared dehydrated with visible wound debris and/or Laponite clay gel within the wound bed. By day 14, the Laponite-VEGF wound was covered with Laponite debris/eschar over the wound, while the alginate-VEGF wound appeared red and moist exhibited a substantial reduction in wound size compared to the alginate vehicle. By day 18, all wounds except the alginate only group had nearly closed with alginate-VEGF exhibited the greatest reduction in wound size but still exhibiting redness.

Figure 6b, c compares would closure for Laponite and alginate, with or without VEGF addition compared to PBS controls. Addition of VEGF to Laponite gels had a negligible effect on wound closure rates compared to Laponite alone. In contrast, compared with alginate only, alginate-VEGF gels exhibited a significant increase in wound closure between 11 and 14, however this difference diminished by day 18. Alginate-VEGF promoted faster wound closure than Laponite-VEGF treatment, particularly between day 11 and 14. PBS wounds displayed faster wound closure than all treatment groups except alginate-VEGF at day 14.

Histological analysis (Fig. 6d) showed similar re-epithelialisation rates between Laponite ± VEGF (Fig. 6e) but significantly faster healing compared to both alginate treatments (Fig. 6f). While VEGF addition significantly increased the rate of re-epithelialisation in alginate, it provided minimal additional improvement over the beneficial effects of Laponite nanoclay alone.

## Discussion

This study demonstrates that a synthetic clay-based biomaterial, Laponite gel, enhances the rate and quality of wound healing in a chronic wound model. We showed that topical Laponite treatment resulted in enhanced re-epithelisation, increased epithelial cell division, and, notably, an induction of hair follicles in the anagen phase in regions close to the wound periphery. This study indicates the potential of clay gels as active agents for delayed wound healing. Although Tegaderm was used for wound coverage, the wound models incorporating strict mechanical controls will allow for a more precise evaluation of Laponite-mediated effects on wound healing in futures studies. As expected, wound closure was delayed in *db/db* mice compared to healthy controls. This difference is the basis for the frequent choice of db/db mice as a model of delayed wound healing. Excisional wounds in wild-type mice heal rapidly through contraction, whereas *db/db* mice primarily rely on re-epithelialization and exhibit less wound contraction compared to normal mice^22^. This is partly due to the thick subcutaneous fat layer in these mice that impairs mechanically the contractile properties of wound myofibroblasts in the healing granulation tissue^26,27^, but is also due to biochemical changes as a result of elevated blood glucose, which are known to lead to a plethora of defects in keratinocyte physiology and function, cytokine deficiencies, impaired stem cell recruitment, low macrophages infiltration, and impaired wound healing^28–32^. While *db/db* mice model cannot fully recapitulate the complexity of chronic non-healing wounds in humans, because of a more natural progression of the disease with prolonged hyperglycaemic states, *db/db* mice represent one of the most widely used and accepted models for studying dysregulated repair in the diabetic context compared with chemical induction^33^.

Although there was no significant difference in the wound closure rate between Laponite-treated and PBS-treated controls in *db/db* mice, we did find a significant increase in re-epithelialization, as measured by epithelial area and thickness. This correlated with a reduction of neutrophils and a marked increase in proliferative cells in the basement membrane of the Laponite-treated group compared to PBS controls.

These results, taken together with evidence of anagen proximal to the wound, suggest that Laponite clay gel promotes the progression from the inflammatory to the proliferative phase of wound healing. The delayed proliferative phase characteristic of chronic wounds is closely linked to the activity of inflammatory cells and fibroblasts^23^. Inflammatory cells, particularly macrophages, play a crucial role in this process, as impaired phenotypic transition of macrophages has been identified as a key factor preventing fibroblast activation and subsequent wound healing in diabetes^34^. Additionally, hyperglycaemia-induced dysfunction of fibroblasts may further contribute to impaired wound healing^35^.

A number of other studies have reported enhanced wound healing using clay-based materials and composites^11–16^ however the mechanism for these effects remain poorly understood. Clays are notable for their high sorptive potential for proteins due to their charged structure and nanoscale size and this is often discussed as a potential mechanism for their healing activity^36,37^. This characteristic makes nanoclays ideal biomolecular delivery vehicles and additives for the sustained release of therapeutic agents and incorporation into composite hydrogels designed for wound healing^2,3,36,38–41^. For example, Ghadiri, et al^36^. intercalated amino acids (arginine, lysine, and leucine) in the lamellar spaces in the structure of Laponite and promote fibroblast proliferation and Page, et al^6^. found that VEGF can be stably incorporated into Laponite gels, effectively sequestering and preserving its activity for prolonged periods in vivo and inducing a higher density of blood vessels compared to Alginate hydrogels^6^.

In the current study, it was notable that, in contrast to alginate gels, addition of VEGF to Laponite clay gels did not promote an enhancement in wound repair over the, already very strong, re-epithelialisation response induced by the clay gel alone. It is possible that Laponite gels may exert effects by stabilising localised concentrations of endogenous growth factors and matrix molecules that promote proliferation and recolonisation, or inversely, by scavenging pro-inflammatory cytokines to attenuate the inflammatory response. Our analysis of clay gel distribution within the wound environment is consistent with both scenarios as a proportion of the clay gel was excluded from the wound while some also remained integrated within the wound bed and the regenerated epithelium.

Laponite has a high surface area and charge distribution that enable strong adsorption of proteins and growth factors, and previous work has shown that VEGF can be retained in Laponite gels in an active form for prolonged periods^6^. We therefore propose that, in the wound environment, Laponite may act not simply as a delivery vehicle for an exogenous growth factor, but as an active biomolecular reservoir that localises and stabilises endogenous host-derived proteins, matrix molecules and growth factors present in wound exudate. Such a mechanism would be consistent with the improved epithelial proliferation, increased epithelial thickness and accelerated re-epithelialisation observed with Laponite alone. Conversely, or additionally, adsorption of inflammatory mediators or proteases by Laponite could reduce their local bioavailability, thereby helping to resolve the prolonged inflammatory phase characteristic of diabetic wounds. This interpretation is consistent with the absence of detectable neutrophils in Laponite-treated wounds at day 18, compared with persistent neutrophil infiltration in PBS-treated controls.

The induction of anagen in regions close to the wound periphery was a striking observation. These findings may suggest that Laponite may modulate the inflammatory phase of wound healing, shortening its duration and facilitating the progression to the proliferative phase to promote hair growth. Hair follicle regeneration has also been observed in a diabetic rat model of wound healing treated with a nanoclay composite gel by Ordeghan, et al^42^. Although the specific contribution of the nanoclay component in this study was not defined. It was notable in the current study that regions of anagen were closely associated with areas of skin in contact with wound exudate and subsequent reddening in these regions. Wound fluid is rich in cytokines and growth factors such as platelet-derived growth factor (PDGF), transforming growth factor-beta (TGF-β), basic fibroblast growth factor (bFGF), tumour necrosis factor-alpha (TNF-α), and various interleukins (ILs) all of which play crucial roles in different stages of wound healing^43,44^. The association of anagen with exposure to wound fluid in nanoclay treatments is suggestive of a role for nanoclay in adsorbing and stabilising localised concentrations cytokines or growth factors in proximity to the skin. Further studies are warranted to test this hypothesis.

In summary, Laponite clay gels offer potential as an active agent for inducing chronic wound healing. While overall wound closure rates were similar between Laponite and PBS treatments, Laponite significantly accelerated re-epithelialisation correlating with reduced inflammation and increased proliferative activity and inducing hair growth at the wound periphery. These findings suggest that Laponite facilitates the transition from the inflammatory to the proliferative phase, a key barrier to healing in chronic wounds. Exploration of the molecular and cellular effects of nanoclay within the immune microenvironment of the chronic wound may be a rich area for future research.

## Methods

### Clay gel preparation

Suspensions of Laponite XLG (BYK Additives, Widnes, UK) were prepared by slowly adding Laponite powder to distilled water under rapid agitation until a vortex was achieved. Suspensions were sterilised by autoclave treatment and weighed pre- and post-sterilisation to calculate water evaporation and % concentrations adjusted accordingly. 3% (w/v) Laponite gel suspensions were used in all experiments. Alginate solutions (1.1% (w/v)) were prepared from anhydrous ultrapure alginate (NovaMatrix, Sandvika, Norway), with UV sterilisation for 30–60 min prior to preparation. To crosslink the alginate gel suspensions, a working solution that contained CaCl_2_ (100 mM) (Merck/Sigma-Aldrich, Gillingham, UK), HEPES (20 mM) (Merck/Sigma-Aldrich) and Tween 20 (0.1 (v/v)%) (Merck/Sigma-Aldrich) was added to a final concentration of 10 mM and mixed thoroughly.

### Animals

All animal procedures were approved by the Animal Welfare and Ethical Review Body (AWERB) of University of Southampton, and conducted in accordance with the UK Animals (Scientific Procedures) Act 1986 and institutional guidelines under Home Office UK Licence PPL 30/2971. The protocols were reviewed and approved by the Faculty Research Ethics Committee (FREC, Medicine) and the University Research Ethics Committee (UREC). All experimental procedures complied with ARRIVE guidelines and institutional animal welfare standards. 18 male C57BL/6 mice (Biomedical Research Facility, University of Southampton) aged between 8 and 10 weeks old were used as a reference for normal wound healing. 37 male *db/db* (BKS.Cg- Dock7^m^ +/+ Lepr^db^/J*)* mice (Envigo, Huntingdon, UK) aged between 8 and 10 weeks old were used to investigate delayed wound healing with treatments (PBS, Laponite, Alginate, Laponite+VEGF, and Alginate+VEGF) (hereafter referred to as *db/db* mice). All animals were bred and maintained at the Biomedical Research Facility, Southampton in a temperature-controlled environment (20–22 °C) with a 12-h light/dark cycle (lights on at 06:00). Food and water were provided ad libitum both pre- and post-injection.

### Wound healing models

**Fig. 7:**
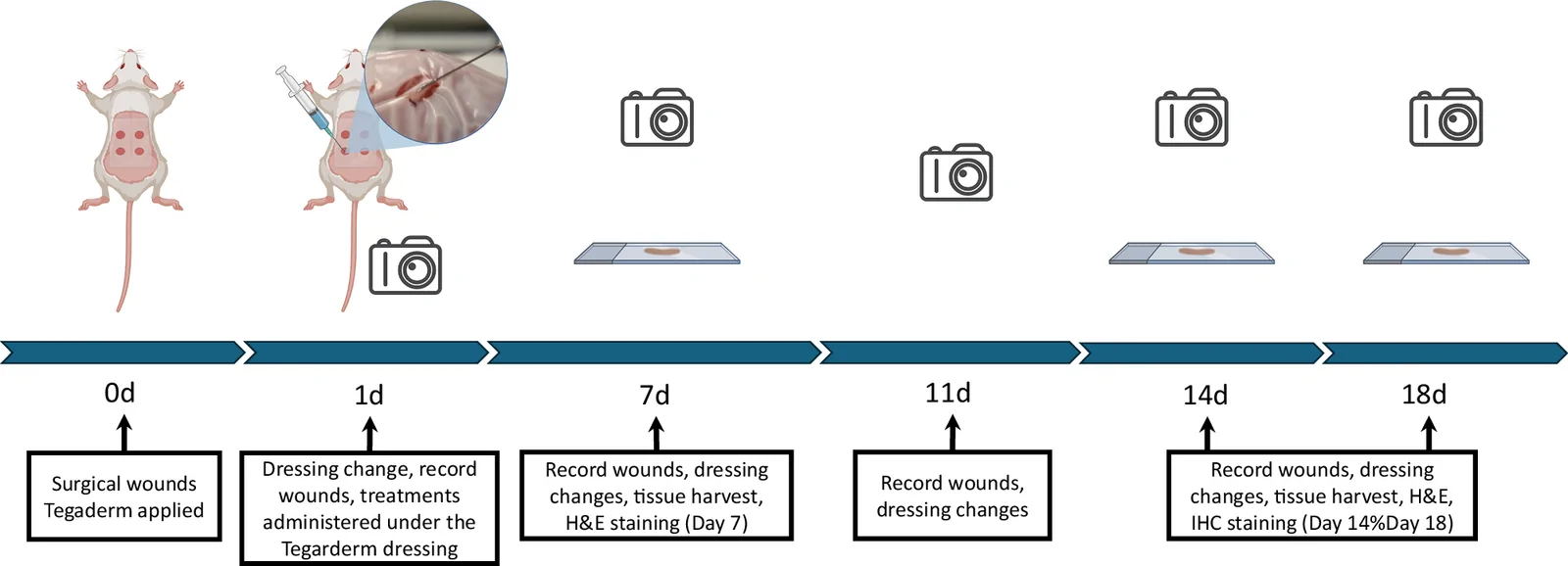
Schematic diagram of overall experimental design (Created by BioRender).

Mice were anesthetized intraperitoneally with 0.4 mg/kg fentanyl citrate and 12.5 mg/kg fluanisone (Hypnorm; VetaPharma Ltd, Leeds, UK) and 12.5 mg/kg midazolam chloride (Hypnovel; Roche, Welwyn, UK). The whole dorsal region of mice was depilated (shaved followed by application of Nair^TM^ hair removal cream (Nair, Ewing, USA)) prior to wounding. A 6-mm diameter biopsy punch and surgical scissors were used to create wounds. In C57BL/6 and *db/db* mice 4 wounds were created dorsally, a pair caudally and a pair rostrally on opposite sides of the mouse. Following surgery, a Tegaderm™ dressing (3 M, Bracknell, UK) was applied over the dorsal region to cover all wounds (dressing was cut to size). Following the procedure, mice were transferred to a pre-warmed (25-30°C) incubator for 2–3 h for recovery, then the mice were removed to room temperature conditions for the remainder of the study. All treatments were administered by injection underneath Tegaderm™ dressing using a 25 G hypodermic needle on day 1 after imaging and redressing. Dressings were changed and wound photographs were taken at different timepoints. For *db/db* mice photographs were taken post-wounding at day 1 (*n* = 9 mice; 18 wounds per group), 7 (*n* = 8 mice; 16 wounds per group), 11, 14 (*n* = 6 mice, 12 wounds per group), and 18 (*n* = 3 mice, 6 wounds per group) using inhaled anaesthetic (isoflurane). Mice were killed by CO2 asphyxiation for 3–5 min followed by cervical dislocation at days 7, 14 and 18. The wound and the surrounding skin were collected at days 7 (*n* = 2 mice; 4 wounds per group), 14 (*n* = 3 mice; 6 wounds per group), and 18 (*n* = 3 mice; 6 wounds per group) after injury and used for subsequent experiments. Wound tissue was fixed in 4% PFA overnight then transferred to 70% (v/v) ethanol and kept in the fridge until required for processing (Fig. 7)^45^. We have indicated the experimental numbers in Supplementary Tables 1 and 2.

### Blood glucose measurements

Non-fasting and fasting blood glucose levels were measured using the Accu-Chek® Aviva Expert Bolus Advisor System (Roche, Burgess Hill, UK) and compatible Accu-Chek® Aviva testing strips (Roche). Blood samples were taken from the tail vein after pricking with a 25 G needle whilst under anaesthetic.

### Macroscopic analysis of wound area, closure rates, and hair follicle anagen area

Wound photographs were captured in situ, after removal of dressing at *T (d)* = *1, 7, 11, 14 and 18* using a Nikon D3200 digital SLR camera and a GoPro Hero 4; a calibrated ruler was included for scaling purposes. The wound area within each image was measured using ImageJ software planimetric analysis to calculate the percentage of wound closure^46^. The area of grey skin colouration surrounding the wound at day 18, confirmed via histological analysis as regions of hair follicle anagen, was also measured on calibrated images using ImageJ planimetric analysis.

### Histological analysis

Fixed tissue samples were dehydrated in graded ethanol and cleared with Histoclear (National Diagnostics, Atlanta, USA) before being embedded in paraffin wax and stored at −20 °C until required for sectioning. Sequential sections were cut at a 10 μm thickness and stored at −80 °C until staining. For H&E staining, histological sections were deparaffinised, rehydrated, and stained with Weigert’s haematoxylin and Eosin (Thermo Fisher Scientific) before mounting with coverslips. For Auramine O staining, sections were incubated with Auramine O solution (Sigma-Aldrich/Merck) for 15 min at room temperature in the dark, washed with distilled water, before mounting with Fluoromount™ (Sigma-Aldrich/Merck). Slides were sealed with nail varnish and stored in the dark until imaging.

### Immunohistochemistry

After being deparaffinised and rehydrated, slide sections were immersed in pre-heated sodium citrate buffer (pH 6.0) at 95–100 °C for 30 min cooled to room temperature washed and then incubated with primary antibodies. Anti-Ki67 (Abcam, 1:500) was incubated at 4 °C for 12–18 before washing and incubation with her secondary antibody (Goat Anti-Rabbit IgG biotinylated, Vector Laboratories, 1:500) for 30 min at room temperature. VECTASTAIN Elite ABC reagent (Vector Laboratories) with 3,3’-diaminobenzidine (DAB) chromogen substrate (Steady DAB/Plus) (Abcam, Cambridge, UK) was used to visualise staining. Negative controls omitting the primary antisera confirmed specificity. For neutrophil marker, anti-myeloperoxidase (anti-MPO) (Abcam; product: ab9535; 1:100 dilution) was applied at the primary antibody step, and applied the same secondary antibody (and dilution factor) that was used for anti-Ki-67 staining. For the semi-quantitative analysis IHC stain, Image J software was used and colour thresholding was applied to detect the percentage of the positive are. Briefly, pixel intensity values for any colour range from 0 to 255, where 0 represents the darkest shade and 255 the lightest. The ROI was set on the area of newly formed epithelial tissue, and the positive areas were quantified from five randomly selected fields within the ROI for the animal sample. Using a consistent threshold setting for all images within the same staining set, the positive staining area was selected, and the percentage of positive staining area was then quantified.

### Measurement of wound closure and rate of re-epithelisation using histological samples

H&E-stained tissue sections scanned by the dotSlide microscope were used to measure the thickness of the wound epithelium. Images were imported within the Olympus Virtual Slide Desktop 2.4 software. Within the Measure→Measurement and ROI window, the Arbitrary line or Polyline tool was selected. Using Arbitrary line tool, the length of the defect was measured from the edge of the wound margin, left to right. Using either Arbitrary line or Polyline tool, the length of epithelisation was then measured (the length of both sides were combined when full re-epithelisation had not occurred). The size of the remaining defect (minus the closure by re-epithelisation) was measured from edge-to-edge of the migrating epithelium.

Histology wound closure rate calculation:

To calculate the change in wound closure rates as a percentage from day 0, the following calculation was used:

1 $$\frac{{{dt}}_{0}-{{dt}}_{x}}{{{dt}}_{0}}\times 100$$

$${Where}:\,{{dt}}_{0}={average\,defect\,size\,at}\,{day}\,0$$

$${{dt}}_{x}={average}\,{remaining}\,{defect}\,{size}\,{at}\,{specific}\,{time}$$

% re-epithelisation rate calculation:

To calculate the rate of re-epithelisation as a percentage increase, this calculation was used:

2 $$\frac{{{ODt}}_{x}}{{{EPt}}_{x}}\times 100$$

Where

$${{ODt}}_{x}={length\; of\; the\; defect\; measured\; from\; the\; edge\; of\; the\; wound\; margin;}$$

$${{EPt}}_{x}={total\; length\; of\; epithelium}$$

### Analysis of wound epithelial thickness

DotSlide-scanned images were imported and measurement tool selected. Using the Arbitrary line tool, the epithelial thickness was measured by drawing a perpendicular line from the surface to the bottom of the epithelium. The number of thickness measurements that were taken across the epithelium was governed by the total length of epithelial tissue present. This number of measurement points were defined as follows: ≤1 mm: 3 points; 1–2 mm: 4 points; 2–3 mm: 5 points and >4 mm 6 points. The measurement points were distributed approximately evenly. This number of points was chosen to enable sufficient distance between measurement points to ensure a regular and evenly spaced distribution of measurements.

### Statistical analysis

A one-way analysis of variance (ANOVA) (parametric) was used for statistical analysis of blood glucose level data and mouse weight data. Tukey’s multiple comparison test was used to determine individual *p* values. A two-way ANOVA (parametric) was used for statistical analysis of wound closure rate data and Tukey’s multiple comparison test was used to determine individual *p* values.

## Supplementary information

Supplementary Data_v4.1.

## Data Availability

All data are available in the main text or the supplementary materials. The raw datasets used and/or analyzed during the current study are available from the corresponding author.
