# Supplementary material for: Nanoclay gels promote re-epithelialization and improve the quality of skin wound healing in a diabetic mouse model
Source: NPJ Regen Med. 2026 Jul 23;11:54. doi: 10.1038/s41536-026-00495-6 (PMC13759475; doi:10.1038/s41536-026-00495-6)
Supplement: Supplementary file 1 [file 41536_2026_495_MOESM1_ESM.pdf]

SUPPLEMENTARY INFORMATION

Nanoclay Gels Promote Re-epithelialization and Improve the Quality of Skin Wound Healing in a Male Diabetic Mouse Model

Daniel J. Page[1,2], Zilian Fan[1] Claire E. Clarkin[3], Rajopal Mani[4], Yang-Hee Kim[1], Jonathan I. Dawson[1], Nicholas D. Evans[1,2*]*

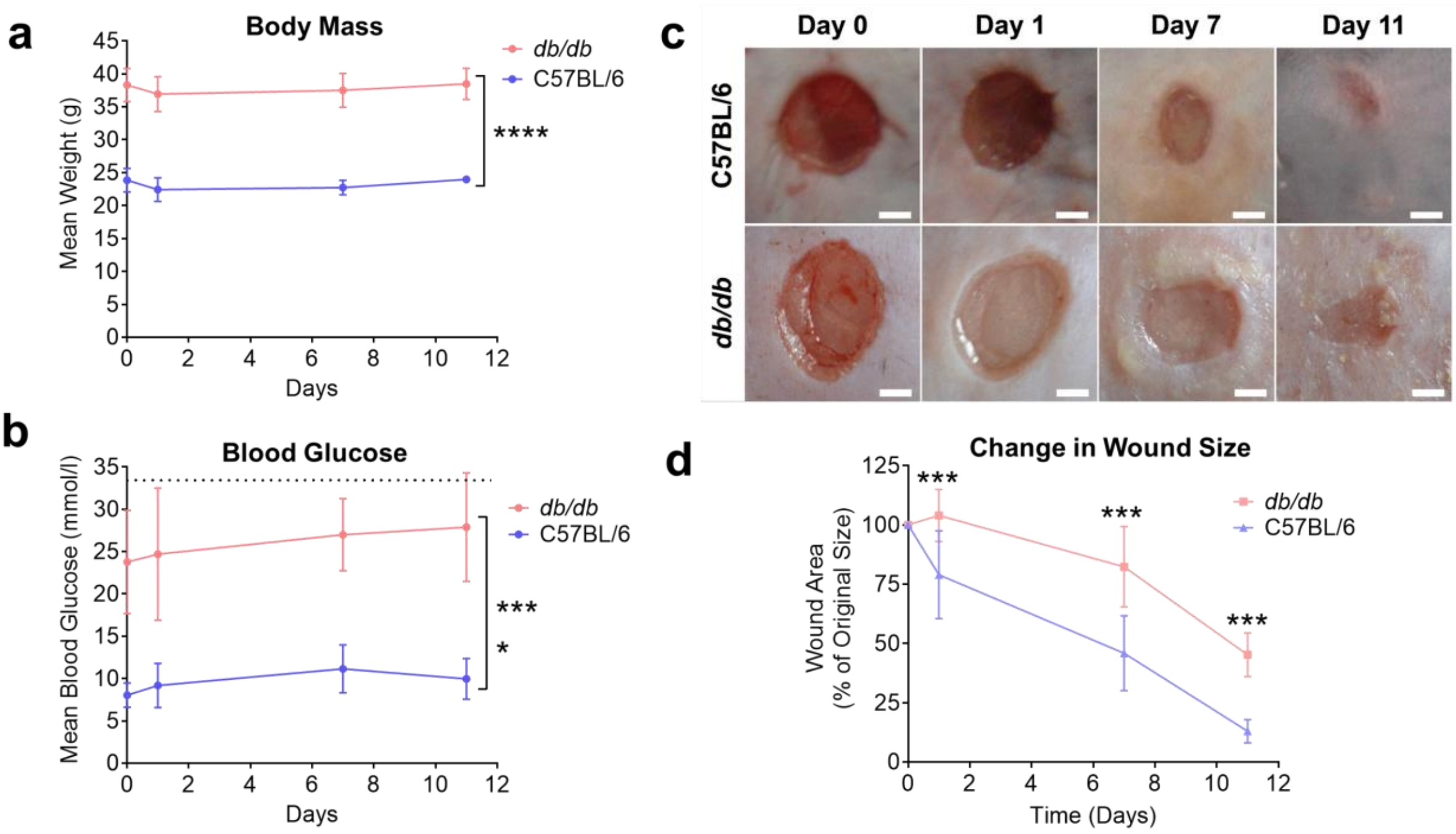

Supplementary Figure 1. Skin wound healing is delayed in *db/db* compared to wild type mice. (a) *db/db* mice weighed significantly more than *wt* mice (~40%) over 11 days (upper panel). C57BL/6 mice=18; *db/db* mice =37. (b) db/db mice exhibited hyperglycaemia over 11 days during the wound healing study period compared to *wt* (lower panel). C57BL/6 mice≤18; *db/db* mice≤37. (c) Macroscopic images show *wt* wounds closed more quickly than *db/db*. Scale bars = 2 mm, which is confirmed (d) quantitatively at all time points up to 11 days. ***P = < 0.001. ****p = < 0.0001. C57BL/6 mice≤6; *db/db* mice≤9. Error bars = SD.

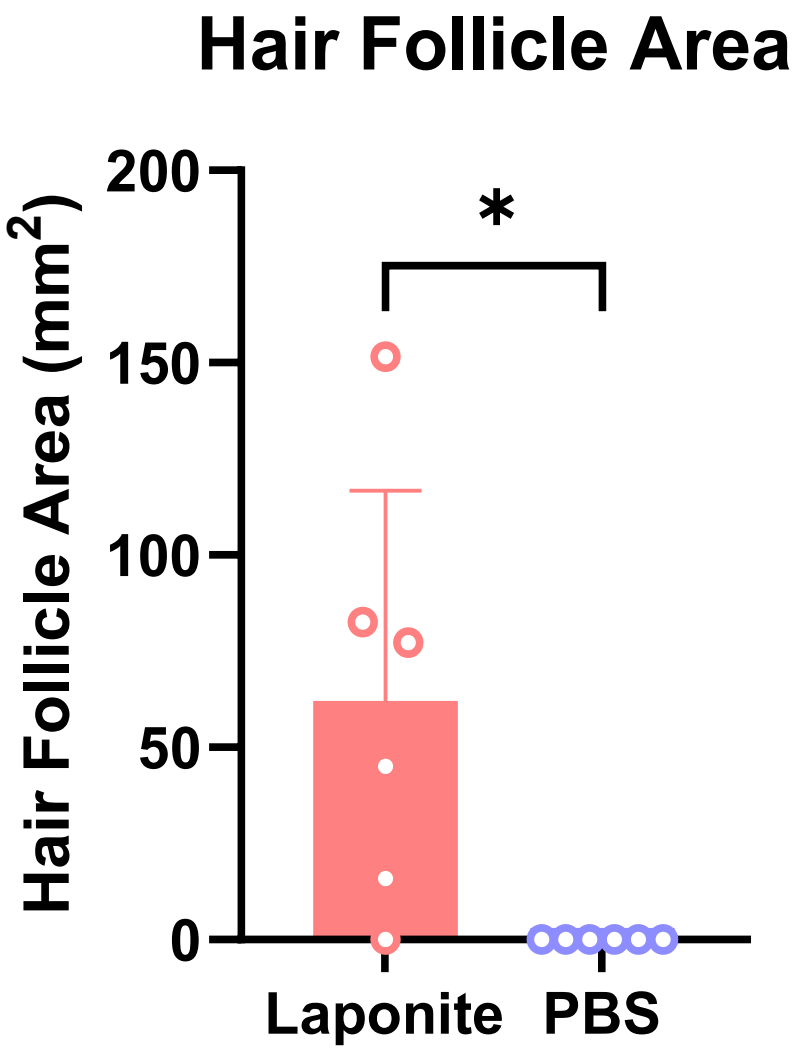

Supplementary Figure 2. Quantification of hair follicle anagen area at Day 18. *P = < 0.05. n=3 (*db/db* mice). Error bars = SD.

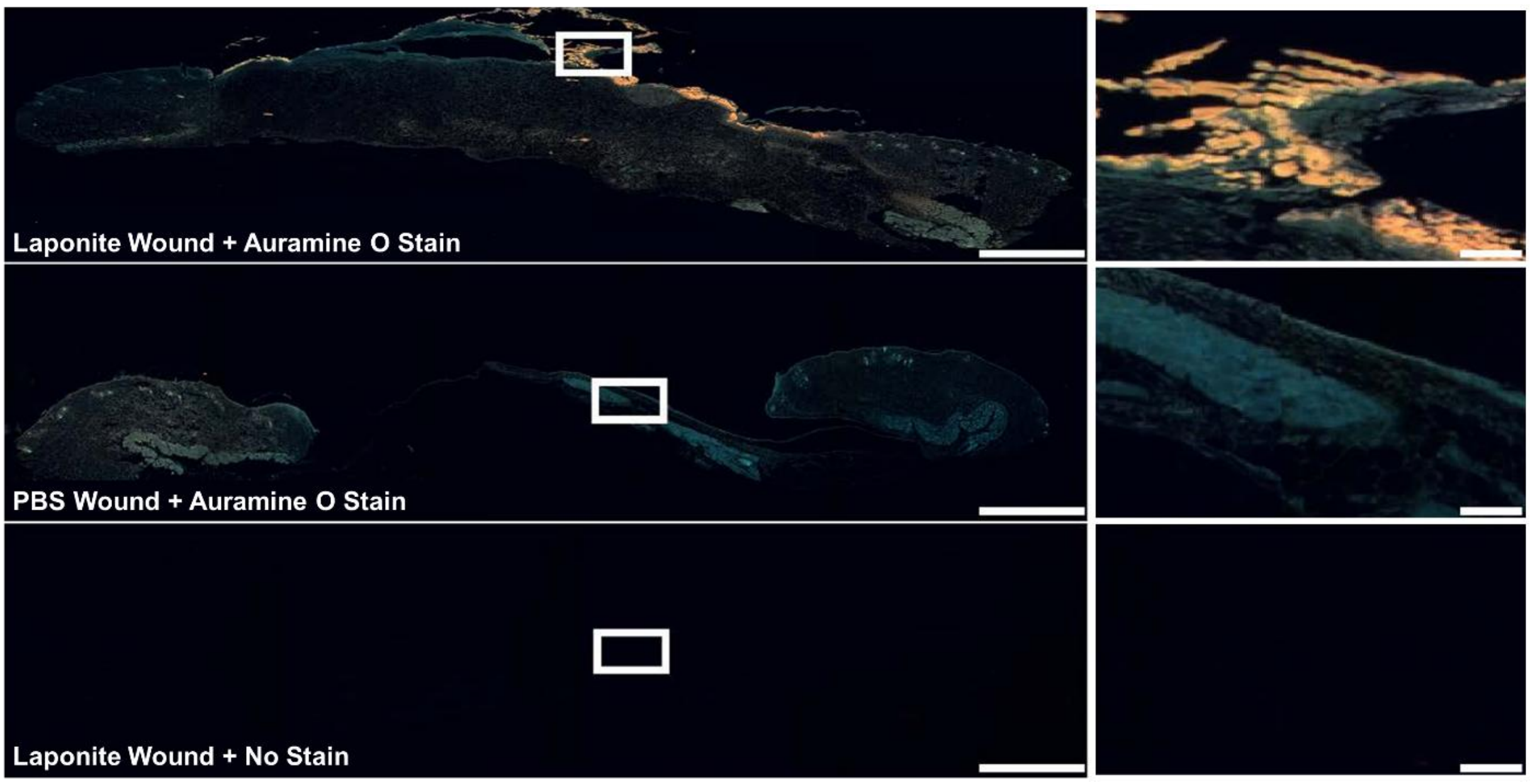

Supplementary Figure 3. Wound tissue stained with Auramine O - a selective marker for Laponite. Wound tissue that had been treated with Laponite hydrogel selectively stained with Auramine O; right panel clearly shows the fluorescent orange stain indicating the presence of Laponite within the wound bed. (B) PBS-treated wound stained with Auramine O; no fluorescent stain present due to the absence of Laponite. (C) Laponite-treated wound with no stain applied used as a control sample. Left panel scale bars = 1 mm, right panel scale bars = 100 µm.

n=mice	C57BL/6	db/db
**Day 0**	16	9
**Day 1**	16	9
**Day 7**	13	8
**Day 11**	8	6
**Day 14**	0	6
**Day 18**	0	3

Supplementary Table 1. The number of animals for the macroscopic wound closure.

n= db/db mice	PBS	Laponite-Vehicle	Laponite-VEGF	Alginate-VEGF	Alginate-Vehicle
**Day 7**	2	2	0	0	0
**Day 14**	3	3	3	3	3
**Day 18**	3	3	3	3	3

Supplementary Table 2. The number of animals per group for histological endpoints.

NOTE: Please save this file locally before filling in the table, DO NOT work on the file within your internet browser as changes will not be saved. Adobe Acrobat Reader (available free here) is recommended for completion.

The ARRIVE guidelines 2.0: author checklist

The ARRIVE Essential 10

These items are the basic minimum to include in a manuscript. Without this information, readers and reviewers cannot assess the reliability of the findings.

Item		Recommendation	Section/line number, or reason for not reporting
**Study design**	1	For each experiment, provide brief details of study design including:<br>a. The groups being compared, including control groups. If no control group has been used, the rationale should be stated.<br>b. The experimental unit (e.g. a single animal, litter, or cage of animals).	Supplementary table 1 and 2<br>Page 19 , Lines 333-335
**Sample size**	2	a. Specify the exact number of experimental units allocated to each group, and the total number in each experiment. Also indicate the total number of animals used.<br>b. Explain how the sample size was decided. Provide details of any *a priori* sample size calculation, if done.	Supplementary table 1 and 2<br>No a priori sample size calculation was performed.
**Inclusion and exclusion criteria**	3	a. Describe any criteria used for including and excluding animals (or experimental units) during the experiment, and data points during the analysis. Specify if these criteria were established *a priori*. If no criteria were set, state this explicitly.<br>b. For each experimental group, report any animals, experimental units or data points not included in the analysis and explain why. If there were no exclusions, state so.<br>c. For each analysis, report the exact value of *n* in each experimental group.	No predefined exclusion criteria were established before the study.<br>No animals or datasets were excluded from the analysis.<br>Supplementary table 1 and 2
**Randomisation**	4	a. State whether randomisation was used to allocate experimental units to control and treatment groups. If done, provide the method used to generate the randomisation sequence.<br>b. Describe the strategy used to minimise potential confounders such as the order of treatments and measurements, or animal/cage location. If confounders were not controlled, state this explicitly.	Page 19<br>randomly treatments and order
**Blinding**	5	Describe who was aware of the group allocation at the different stages of the experiment (during the allocation, the conduct of the experiment, the outcome assessment, and the data analysis).	Investigator did not know the treatments
**Outcome measures**	6	a. Clearly define all outcome measures assessed (e.g. cell death, molecular markers, or behavioural changes).<br>b. For hypothesis-testing studies, specify the primary outcome measure, i.e. the outcome measure that was used to determine the sample size.	Done
**Statistical methods**	7	a. Provide details of the statistical methods used for each analysis, including software used.<br>b. Describe any methods used to assess whether the data met the assumptions of the statistical approach, and what was done if the assumptions were not met.	In Methods part<br>Page 23
**Experimental animals**	8	a. Provide species-appropriate details of the animals used, including species, strain and substrain, sex, age or developmental stage, and, if relevant, weight.<br>b. Provide further relevant information on the provenance of animals, health/immune status, genetic modification status, genotype, and any previous procedures.	Page 18
**Experimental procedures**	9	For each experimental group, including controls, describe the procedures in enough detail to allow others to replicate them, including:<br>a. What was done, how it was done and what was used.<br>b. When and how often.<br>c. Where (including detail of any acclimatisation periods).<br>d. Why (provide rationale for procedures).	Page 19
**Results**	10	For each experiment conducted, including independent replications, report:<br>a. Summary/descriptive statistics for each experimental group, with a measure of variability where applicable (e.g. mean and SD, or median and range).<br>b. If applicable, the effect size with a confidence interval.	Done
